# Una relación singular: Los vínculos entre gestantes y comitentes de gestación por sustitución entre Ucrania y Argentina

**DOI:** 10.18294/sc.2025.5347

**Published:** 2025-04-03

**Authors:** Lucía Ariza, Natacha Salomé Lima

**Affiliations:** 1 Doctora en Sociología. Investigadora Asistente, Consejo Nacional de Investigaciones Científicas y Técnicas, con sede en Instituto de Investigaciones Gino Germani, Universidad de Buenos Aires, Ciudad Autónoma de Buenos Aires, Argentina. lucia.ariza@gmail.com Consejo Nacional de Investigaciones Científicas y Técnicas Consejo Nacional de Investigaciones Científicas y Técnicas Instituto de Investigaciones Gino Germani Universidad de Buenos Aires Ciudad Autónoma de Buenos Aires Argentina lucia.ariza@gmail.com; 2 Doctora en Psicología. Investigadora Asistente, Consejo Nacional de Investigaciones Científicas y Técnicas, con sede en Facultad de Psicología, Universidad de Buenos Aires, Ciudad Autónoma de Buenos Aires, Argentina. lima.natacha@hotmail.com Consejo Nacional de Investigaciones Científicas y Técnicas Consejo Nacional de Investigaciones Científicas y Técnicas Facultad de Psicología Universidad de Buenos Aires Ciudad Autónoma de Buenos Aires Argentina lima.natacha@hotmail.com

**Keywords:** Salud Reproductiva, Gestación por Sustitución, Técnicas Reproductivas Asistidas, Argentina, Reproductive Health, Gestational Surrogacy, Assisted Reproductive Techniques, Argentina

## Abstract

La gestación por sustitución es una práctica que genera controversias, especialmente en contextos donde la regulación es escasa o inexistente. En Argentina, aunque no se encuentra legislada, esta práctica cuenta con significativa intermediación judicial y diversas estrategias legales para su consecución. Las inseguridades jurídicas locales han llevado a muchas familias argentinas a optar por realizar procedimientos de gestación por sustitución en otros países, siendo Ucrania un destino destacado por su accesibilidad y características específicas. Este estudio tiene por objetivo analizar, desde la perspectiva de las comitentes, las relaciones entre las mujeres cisheterosexuales casadas que buscan ser o han sido madres (comitentes) a través de gestación por sustitución y las gestantes ucranianas. Desde un diseño cualitativo-exploratorio, entre 2022 y 2024, se realizaron 18 entrevistas en profundidad a personas que hubieran realizado gestación por sustitución y que se hubieran desplazado geográficamente para ello. Los resultados muestran que los vínculos establecidos trascienden las geografías y se nutren de un intercambio bidireccional de objetos (fotos, regalos, canciones, entre otros) y emociones, configurando relaciones singulares. Estos hallazgos permiten complejizar los análisis de la gestación por sustitución, alejándose de las visiones simplistas que la reducen a la instrumentalización del cuerpo de las mujeres, y destacan las dimensiones relacionales y afectivas de estas experiencias.

## Abstract (en)

Surrogacy is a practice that generates controversy, especially in contexts where regulation is scarce or nonexistent. In Argentina, although there is no specific legislation, this practice involves significant judicial mediation and various legal strategies for its implementation. Local legal uncertainties have led many Argentine families to pursue surrogacy procedures in other countries, with Ukraine standing out as a prominent destination due to its accessibility and specific characteristics. This study aims to analyze, from the perspective of intended parents, the relationships between married cisheterosexual women who seek or have become mothers (intended parents) through surrogacy and Ukrainian gestational carriers. Using a qualitative-exploratory design, between 2022 and 2024, 18 in-depth interviews were conducted with individuals who had undergone surrogacy and traveled internationally for the process. The results show that the established bonds transcend geographical boundaries and are enriched by a bidirectional exchange of objects (photos, gifts, songs, among others) and emotions, shaping singular relationships. These findings contribute to a more nuanced understanding of surrogacy, moving beyond simplistic views that reduce it to the instrumentalization of women’s bodies and highlighting the relational and affective dimensions of these experiences.

## INTRODUCCIÓN

En la literatura socioantropológica, el término “gestación por sustitución” refiere a un arreglo reproductivo a través del cual una persona (usualmente denominada gestante) lleva intencionalmente adelante un embarazo para otra persona o pareja (usualmente denominada comitente), que será legalmente reconocida como progenitora o progenitor del bebé así nacido^1^^,^^2^^,^^3^. Mientras que la llamada “gestación por sustitución *tradicional*” implicaba una gestación en la que se utilizaban los propios óvulos de la gestante, la expansión de las técnicas de reproducción humana asistida ha permitido el desarrollo de la subrogación *gestacional* (“*gestational surrogacy*” en inglés)^1^^,^^2^; es decir, aquella en la que gestante y bebé no comparten genética. En Argentina y otros países como Ucrania y algunos estados de EEUU, entre otros, las guías bioéticas y regulaciones existentes aconsejan y/o reconocen como únicamente válida la gestación por sustitución sin vínculo genético entre gestante y bebé^4^^,^^5^.

A medida que se desestabilizan nociones y experiencias firmemente asentadas en Occidente respecto de la continuidad entre embarazo y maternidad, la gestación por sustitución constituye un fenómeno complejo y controvertido que revela tensiones profundas entre las posibilidades ofrecidas por las tecnologías reproductivas y los condicionamientos normativos, culturales y bioéticos que enfrentan las personas involucradas en estos procesos. Asimismo, teniendo en cuenta que, en el mundo occidental, la asociación entre dinero y cuerpo suele ser depositaria de sentidos negativos^6^^,^^7^, el hecho de que los procesos de gestación por sustitución pueden estar intermediados por dinero, supone una capa adicional de complejidad para entender el fenómeno de la gestación por sustitución en Argentina y el resto del mundo occidental^5^^,^^8^. De allí deriva también el carácter disputado de la gestación por sustitución como *derecho reproductivo*: por un lado, desde diferentes visiones se sostiene que promueve el derecho a decidir y a la mater/paternidad del colectivo LGTB y de personas con dificultades reproductivas, por otro, se sostiene que restringe el derecho de las gestantes a abortar o decidir sobre su propio cuerpo^2^, lo que es fuente de fuertes controversias. 

En Argentina, la gestación por sustitución no está regulada ni está prohibida, lo que genera muchas inseguridades jurídicas para las personas que no tienen otra alternativa que esta para tener descendencia. Para algunas personas, esta ausencia legislativa es motivo de búsqueda de opciones reproductivas fuera del país. Así, Ucrania, que era inicialmente visualizado como uno de los destinos más inhóspitos, comenzó con el tiempo a ser percibido como el que más garantías brindaba a la hora de iniciar un proceso de gestación por sustitución. 

Esta investigación, que se inscribe en un proyecto en curso sobre movilidades reproductivas, buscó conocer las experiencias de las personas que viajaron a Ucrania para iniciar un proceso de gestación por sustitución. El estudio buscó indagar cómo y por qué se decidieron por esta opción reproductiva, por qué eligieron realizarla en ese país, cómo seleccionaron a la donante de óvulos, si tuvieron o no vinculación con la gestante y de qué tipo, qué relación mantuvieron con Ucrania como país de gestación de su descendencia, cómo transmitieron la información sobre la realización de una gestación por sustitución a sus seres cercanos, cómo pensaban transmitirle el relato de sus orígenes a sus hijos y/o hijas, entre otros.

En este artículo presentamos resultados de un aspecto particular de esta indagación, que son las expectativas y deseos de las comitentes argentinas de conocer a las gestantes ucranianas antes de viajar a Ucrania, así como las relaciones que mantuvieron a lo largo del embarazo, inmediatamente después y/o más adelante, una vez que habían regresado a Argentina con sus hijos o hijas. Nuestro interés es dar cuenta de las maneras en las cuales la realización de un procedimiento, como es la gestación por sustitución, implica la generación de un vínculo, real o imaginario, entre quienes buscan convertirse en madres a través de esta opción reproductiva, y quienes ofrecen gestar ese embarazo a cambio de una retribución económica (como es la norma en todos los casos de la gestación por sustitución en Ucrania). Buscamos así producir datos y análisis de investigación empírica sobre este tipo de arreglo reproductivo desde una perspectiva socioantropológica usualmente en falta en los álgidos debates concernientes a la gestación por sustitución, como también aportar una mirada que complejice y enriquezca las caracterizaciones simples de la gestación por sustitución como una práctica que “instrumentaliza” el cuerpo de las mujeres y convierte en mercancía el inalienable trabajo reproductivo, así como a los niños y las niñas que nacen por gestación por sustitución^9^^,^^10^^,^^11^. 

La cuestión de qué tipo de relación se entabla entre comitentes y gestantes, a través de qué medios, con qué expectativas y afectos es especialmente relevante en el marco de este proyecto debido a la distancia que separa a Ucrania de Argentina y a las movilidades reproductivas necesariamente involucradas en esta gestación. En efecto, mientras que en todos los casos de gestación por sustitución -ocurran o no en el mismo país y se trate o no de una práctica remunerada- existe algún tipo de vínculo -por más remoto, abstracto o administrativo que sea- entre comitentes y gestantes, cuando esta práctica se realiza a través de distancias geográficas muy acuciantes, como es en este caso, la cuestión del vínculo adquiere un factor crucial. Como mostraremos en este escrito, la conexión que se establece entre progenitores de intención y gestantes es, a su vez, bidireccional y mutuamente buscada, lo que nos permite deshacer las connotaciones de instrumentalidad, comercio e incluso extractivismo con que a veces se critica a la gestación por sustitución^12^^,^^13^^,^^14^^,^^15^^,^^16^. 

Este artículo es parte del proyecto “Repro-flujos en Europa, África del Norte y América Latina: la movilidad de personas y gametos en el contexto fragmentado de la regulación transnacional en adopción y reproducción asistida” (PID2020-112692RB-C21 / AEI / 10.13039/501100011033) y ha sido financiado por el Ministerio de Ciencia e Innovación de España.

### Nuevas figuras: nominaciones en disputa

Respecto a los términos con los que nos referimos aquí a las y los actores del proceso de gestación por sustitución, utilizamos la expresión “comitentes” para aludir a progenitores de intención que buscan mater-paternar a través de esta opción reproductiva. Consideramos que si bien el término no deja de mostrar cierta problematicidad inherente al nombrar a quienes buscan convertirse en madres y padres como las y los principales artífices del proceso -cuando en realidad, como indican Gunnarsson Payne *et al*.^2^, la gestación por sustitución es posible gracias a la intervención de una serie mucho más larga de agentes, dentro de la que las y los progenitores son solo un eslabón, aunque central- es justamente este rasgo el que, políticamente hablando, resulta significativo. En efecto, el término “comitentes” permite despejar dudas respecto de quién ejercerá efectivamente el rol de madre o padre, al entroncar con la “voluntad procreacional” como piedra angular para la filiación por tecnologías reproductivas^17^^,^^18^^,^^19^. 

En Argentina, este criterio ha sido sancionado en el nuevo Código Civil y Comercial de la Nación^20^ que, si bien dejó sin legislar la gestación por sustitución, en el Artículo 562 definió la “voluntad procreacional” como aquella que produce filiación entre:

…los nacidos por las técnicas de reproducción humana asistida [y] del hombre o de la mujer que *también* ha prestado su consentimiento previo, informado y libre en los términos de los artículos 560 y 561, debidamente inscripto en el Registro del Estado Civil y Capacidad de las Personas, con independencia de quién haya aportado los gametos.^20^^)^

En ocasiones, y a los efectos de evitar repeticiones, recurrimos a los términos madres/padres/progenitores de intención para referirnos a las y los comitentes.

A lo largo de este texto utilizamos el concepto de “gestación por sustitución” en lugar de otros términos alternativos usados frecuentemente, como “gestación subrogada”, “maternidad subrogada” o “subrogación (de vientre)” o, en vertientes críticas de la gestación por sustitución, los de “vientre de alquiler”, “alquiler de útero”, “maternidad de alquiler”, “maternidad por encargo”, entre otros^21^. Por un lado, el concepto de “gestación subrogada”, u otros que involucren algún término de la familia de palabras de “subrogación”, no serían semánticamente adecuados ya que no se adecuarían a la definición de la Real Academia Española (RAE) del verbo “subrogar”: “sustituir o poner a alguien o algo en lugar de otra persona o cosa”^22^, ya que en la gestación por sustitución no se sustituye la totalidad de los procedimientos involucrados en la reproducción (como el sexo, o el aporte genético)^21^. Sin embargo, frente a esta crítica, cabe aclarar que es la propia RAE la que ofrece la definición de “gestación subrogada” como el “embarazo en que una mujer gesta un embrión ajeno”^23^ y, de hecho, el término “gestación por sustitución” es referenciado a aquel. 

Por otro lado, dadas las connotaciones comerciales del término “alquilar”, definido como “tomar de alguien algo para usarlo por el tiempo y precio convenidos” o “dicho de una persona: ponerse a servir a otra por cierto estipendio”^24^, es claro que expresiones como “vientre de alquiler” o “alquiler de útero”, que buscan enfatizar el aspecto eventualmente comercial de la gestación por sustitución, dejan afuera a todos aquellos casos en los que no existe un intercambio de dinero a cambio de la gestación, a la vez que incluyen un tinte crítico en el propio término. Por último, todas las modalidades que implican derivados del término “maternar” son equivocadas, dado que la mujer que gesta en la gestación por sustitución no materna. Por esta razón, nos decidimos por el uso del término “gestación por sustitución”, el que parece estar a salvo de los aspectos más problemáticos de las otras formas de nombrar la práctica reproductiva. Por esta misma razón, nos referimos a quienes han llevado adelante los embarazos como gestantes, y no como “madres”, ni “madres subrogadas” o “madres alquiladas”.

### Dimensión relacional, altruismo e intercambio monetario

Para este estudio tomamos dos grupos de antecedentes. Por un lado, recuperamos estudios previos que han indagado en las relaciones mutuas que entablan personas que atraviesan y/o participan en la consecución de procedimientos de gestación por sustitución, incluyendo progenitores de intención, gestantes, niños y niñas nacidos de estos procedimientos, etc., así como en el aspecto característicamente *relacional*, *distribuido y colaborativo* de la gestación por sustitución. Por otro lado, ponemos en discusión antecedentes que han tematizado la cuestión del altruismo y/o alternativamente, del intercambio monetario, como marco para la realización de gestación por sustitución.

Respecto del primer punto, resultan particularmente relevantes las investigaciones que han dado cuenta de la gestación por sustitución como una práctica que, más allá de escenificar de manera central la cuestión de la autonomía y los derechos reproductivos supone también el establecimiento de relaciones de colaboración entre personas, lo cual incluye, en la gestación comercial, el intercambio de dinero. Estas relaciones complejizan la noción de agencia, que más que emanar paradigmáticamente del sujeto liberal como su única fuente, revelan el carácter entramado de las relaciones entre los diferentes actores de la gestación por sustitución. En esta línea, nos interesa rescatar, siguiendo a Anika König^3^, la noción propuesta por Rayna Rapp^25^ de “enredos [*entanglements*] reproductivos” para describir la complejidad de los mundos sociales a través de los cuales se da la innovación e intervención tecnológica en el ámbito reproductivo. Los enredos reproductivos refieren, entre otras cosas, a los nuevos tipos de relaciones y de actores que se producen a través de la innovación tecnológica, y apuntan a enfatizar que las intervenciones novedosas en el ámbito de la reproducción necesitan sedimentarse a través de una serie de actores a lo largo de los cuales la acción se halla *distribuida*. Este punto ha sido también enfatizado por Gunnarsson Payne *et al*.^2^, quienes refieren a la gestación por sustitución como una forma de “reproducción *colaborativa*”^2^, e indican que el “aspecto relacional” de la gestación por sustitución, incluyendo los estudios de los efectos sobre la gestante, los progenitores de intención y los niños y niñas que nacen continúan siendo notablemente pocos.

Algunos de los trabajos que han indagado específicamente en estas relaciones son, por ejemplo, los producidos por investigadoras del Centre for Child, Adolescent and Family Research de la Cambridge University. Los estudios de Jadva e Imrie^1^ y Jadva, Imrie y Golombok^26^ examinaron las relaciones que mantenían las gestantes con las comitentes y con las niñas y los niños que habían nacido, luego de algunos años de ocurrida la gestación por sustitución. Si bien estos estudios miraron tanto las prácticas de gestación por sustitución tradicional (donde se usan los óvulos de la gestante) y gestación por sustitución sin vínculo genético entre gestante y bebé, las autoras arribaron a la conclusión de que la mayoría de las gestantes deseaban tener y mantenían buenos vínculos con las familias comitentes luego de varios años de producida la gestación, independientemente del tipo de gestación realizada. Estos estudios indagaron también en las maneras en las cuales tanto las gestantes y sus familiares (hijos, hijas, parejas) como las familias formadas a partir de la gestación por sustitución, buscaban utilizar alguna terminología que pudiera dar cuenta del rol especial que había jugado la gestante en esa gestación. Por ejemplo, los niños y las niñas que habían nacido por gestación por sustitución solían llamar a sus gestantes utilizando terminología de parentesco, como “madrinas”, “tías” o “mamás [de] panza [*tummy mummy*]”, un punto que también han mostrado Cadoret^27^ y Olavarría^28^, y en muchos casos las gestantes referían a las niñas y los niños que nacían gracias a su gestación como “sobrinos o sobrinas”. Otra terminología referida ha sido también la de “*surrosister*” o “*surrobrother*”^1^ que hablan de la particular modalización de una terminología de parentesco para nombrar a las nuevas relaciones nacidas con la gestación por sustitución.

Gestar para otros supone una relación íntima que alude a lo familiar y, por ello, a lo endógeno, pero al ser realizada por una persona extraña o ajena al proyecto parental, también supone lo exógeno, un aspecto que ha sido más enfatizado en las investigaciones de Olavarría^28^. Por su parte, el psicoanálisis lacaniano identifica a este tipo de fenómenos con el neologismo de lo éxtimo, que remite al *unheimlich* freudiano^29^, es decir, aquello inquietantemente familiar y extraño a la vez. Los vínculos que producen las biotecnologías reproductivas asocian lo íntimo, lo doméstico, con el intercambio en el marco de un trabajo reproductivo compartido y acordado, pero también, en muchos casos, remunerado. Este último factor, el intercambio económico, sumado a la intervención tecnológica, incluye nuevos elementos en el entorno familiar, cuyos sentidos comunes en el Occidente moderno están disociados de la economía de mercado y de la intervención biotecnológica.

Esta última observación nos conecta con el segundo cuerpo de antecedentes que registramos, y que tematizan la cuestión del altruismo y/o alternativamente, del intercambio monetario, como marco para la realización de gestación por sustitución. Este aspecto ha sido abordado, por ejemplo, desde las coordenadas del trabajo emocional y del trabajo relacional^30^^,^^31^^,^^32^^,^^33^, enfoque elegido en estudios anteriores que exploraron el contacto socioafectivo y las relaciones familiares entre gestantes y parejas españolas que recurrieron a la gestación por sustitución en EEUU, Canadá y Ucrania. De diferentes maneras, estas autoras mostraron cómo el contexto social modela la actuación de las gestantes, desde lo que se espera de ellas como parte de las narrativas biomédicas, hasta el contexto más amplio que puede involucrar mandatos y expectativas religiosas de sus comunidades. Así, las motivaciones para gestar se vuelven inteligibles desde los significados socialmente compartidos que influyen los sentimientos y comportamientos individuales^34^. En este sentido, incluso aquellas gestantes que se han profesionalizado, es decir, que han transformado su experiencia vivida como gestante en competencias para la inserción en la industria de la fertilidad -por ejemplo, convirtiéndose en empleadas o *managers* de agencias de subrogación en EEUU- aluden a su trabajo, sorteando campos ideológicos y marcos normativos aparentemente opuestos (altruismo vs. comercio) al utilizar oximorones denominativos como “trabajo de amor”^33^^,^^35^. 

En efecto, mientras que la empatía y el altruismo han sido identificadas como motivaciones primarias para la participación de gestantes que viven en los países centrales^36^, en el caso de otros contextos más periféricos, la participación en un proceso de gestación por sustitución puede constituirse más directamente en un medio de emancipación o incluso de ascenso o mejora social que no podría ser alcanzado de otro modo, como es el caso de Tailandia^37^^,^^38^. 

En América Latina, prima una condición particular. Mientras es claro que muchas mujeres que ofician de gestantes lo hacen desde posiciones subalternas y, por lo tanto, el interés económico es frecuente, al mismo tiempo existe una fuerte presión para caracterizar los arreglos de gestación por sustitución, al menos nominalmente, como altruistas. Este es el caso de México, donde la retórica del altruismo profundiza aún más las desigualdades previas^39^, desprotegiendo e imponiendo condiciones de asimetría que evidencian el sistema de la reproducción estratificada^40^. En el caso de Argentina, mientras la gestación por sustitución ocurre en la mayoría de los casos a través de intercambios económicos, existe una presión bioética, legal y moral en caracterizarla como “solidaria” y borrar rastros del intercambio monetario, produciendo un doble estándar^41^. En otros países de la región, por su parte, la gestación por sustitución no está permitida a menos que sea altruista y llevada a cabo cuando la gestante y los padres de intención mantienen una relación de consanguinidad, como es el caso de Brasil^42^ o Uruguay^43^. Esto demuestra que, en algunos países de la región, el entramado normativo prefiere acuerdos intrafamiliares bajo el supuesto de que al promover la solidaridad y el altruismo se evitarían las consecuencias no deseadas del intercambio monetario, como la posible explotación de las mujeres durante estos procesos. 

En el contexto latinoamericano, por lo tanto, e independientemente de si hay intercambio monetario o no, se nota el esfuerzo por mantener a la gestación por sustitución en la órbita de lo familiar y lo altruista. En otras partes del mundo, el giro hacia acuerdos altruistas y/o familiares, como medio para suprimir los conflictos existentes, produjo cambios en las reglamentaciones, principalmente cerrando las fronteras para extranjeros, como es el caso de India^44^^,^^45^^,^^46^ o Tailandia^37^^,^^38^^,^^47^^,^^48^. Sin embargo, los estudios muestran^49^ que las situaciones de las mujeres gestantes no han cambiado y, en algunos casos, han empeorado. Estas dinámicas propias del Sur Global han seguido también patrones similares en el contexto méxicano^50^. Investigaciones etnográficas en ese país^39^^,^^51^ demuestran el impacto en las prácticas, producto de un ambiente de semiclandestinidad y desconfianza. 

## METODOLOGÍA

Dado el escaso conocimiento producido hasta ahora en Argentina y la región en lo relativo a las movilidades reproductivas y la gestación por sustitución, esta investigación tuvo un diseño cualitativo-exploratorio. El estudio se propuso conocer de manera amplia cuáles fueron las experiencias de las personas que decidieron realizar un procedimiento de gestación por sustitución y que tuvieron que desplazarse geográficamente para realizarlo, ya sea a Ucrania u otros lugares de Argentina. 

A partir de un primer contacto personal de una de las investigadoras, a través del cual se pudo entrevistar a una mujer que había realizado gestación por sustitución en Ucrania, se comenzó a contactar a otras potenciales entrevistadas utilizando la técnica de “bola de nieve”^52^. Este reclutamiento dio lugar a una muestra conformada por 18 personas, de las cuales 17 eran mujeres cisheterosexuales que habían viajado a Ucrania, mientras que solo una de ellas había realizado la gestación por sustitución en Argentina. Por lo tanto, mientras el diseño original del estudio no restringía la participación a mujeres ni a personas que hubiesen ejercido movilidades reproductivas en otros países, el uso de la técnica de bola de nieve -idóneo, dado el carácter sensible de la temática y estadísticamente reducido y difícil de localizar del sujeto de estudio^53^-, implicó que la muestra quedara exclusivamente conformada por mujeres que en su mayoría habían viajado a Ucrania (salvo una), sin la presencia de varones (con la excepción de una entrevista en la que, si bien fue conducida con la mujer, su pareja y padre del bebé concebido por gestación por sustitución en Ucrania estaba también presente y realizó algunas acotadas intervenciones). A su vez, en tanto en Ucrania la gestación por sustitución solo es legal para el caso de parejas cisheterosexuales casadas^5^, este fue otro determinante para que la muestra no estuviese compuesta por otras identidades sexuales o de género. Dado el carácter difícil de este trabajo de campo, se optó por aceptar las referencias espontáneas de las entrevistadas, evitando sugerir que estuviesen también presentes sus parejas varones.

Las entrevistas analizadas en este artículo se realizaron entre enero 2022 y mayo 2024. El trabajo de campo coincidió temporalmente con la invasión ucraniana por parte de Rusia, una particularidad que, si bien no podemos abordar en su gran complejidad en este artículo debido a falta de espacio, mencionaremos cuando sea oportuno desde el punto de vista de nuestros análisis. Cabe aclarar que la coincidencia temporal con la guerra entre Rusia y Ucrania no implica que todos los casos recopilados estuviesen afectados por este evento, ya que muchas mujeres entrevistadas ya habían tenido sus hijos en Ucrania antes de la invasión rusa. 

Más allá de favorecer una aplicación flexible que, de acuerdo a los preceptos de la investigación cualitativa, evitara romper el flujo de la conversación y el interés de las participantes^53^, la guía de preguntas estuvo estructurada en tres grandes secciones cronológicas: antes, durante y después de la gestación por sustitución. En la primera sección, se buscó conocer aspectos de la historia reproductiva y de la búsqueda de descendencia de la mujer y la pareja, y su vínculo con la decisión de realizar gestación por sustitución. Se indagó en las percepciones sobre las diferencias entre realizar gestación por sustitución en Argentina y realizarla en otro país, y una vez que se comenzaron a realizar entrevistas con mujeres que habían viajado a Ucrania, se preguntó por los motivos para buscar realizar gestación por sustitución fuera de Argentina y para elegir Ucrania, en particular, como destino. También se preguntó por las visiones de las entrevistadas respecto de la gestación por sustitución en general (por ejemplo, si sabían de qué se trataba antes de empezar a considerarla como opción y qué opiniones tenían sobre la gestación por sustitución previo a realizarla), así como por nociones sobre la importancia del vínculo genético para la formación de vínculos de parentesco. 

En la segunda sección, se indagó sobre la forma de selección y vinculación con la clínica a cargo de la gestación por sustitución, incorporando preguntas sobre la relación con la clínica ucraniana a medida que se desarrollaba el trabajo de campo. También se preguntó en profundidad sobre el proceso de elección de la donante (en los casos en que se utilizó donación de óvulos), proceso que no suele estar permitido en Argentina. Este punto no estaba previsto en la guía de preguntas, pero fue incluido una vez que se observó, con el trabajo de campo, que en muchos casos las personas comitentes habían podido seleccionar a la donante de óvulos. Se buscó recolectar información sobre la relación con la gestante (en los casos en que la hubo) durante el embarazo, así como las consideraciones respecto del intercambio monetario y las formas de retribución que muchas entrevistadas utilizaron para agradecer a la gestante más allá del dinero. Este último punto fue un elemento emergente que se profundizó a medida que se realizaba el trabajo de campo e iba quedando claro que los intercambios económicos no agotaban la relación de retribución y agradecimiento que las personas comitentes entablan en muchos casos con la gestante. También se indagó, cuando fue posible o relevante, en los deseos o expectativas que tenían las madres entrevistadas de conocer o no a la gestante, o de entablar un vínculo con ella. 

En la tercera sección, se preguntó sobre las emociones e impresiones luego del primer encuentro con la o el bebé y, en los casos en que ocurrió, con la gestante; los planes para transmitir los orígenes a la descendencia, las experiencias de discriminación o estigma que las participantes hubieran sufrido como consecuencia de haber decidido realizar gestación por sustitución, entre otros aspectos de relevancia. Con algunas entrevistadas que tenían una gestación por sustitución en curso en Ucrania al iniciarse la guerra con Rusia, en febrero 2022, se profundizó también en esta experiencia, la cual supuso un conjunto de desafíos específicos que afectaron profundamente el transcurso del procedimiento de gestación por sustitución, los cuales, por razones de espacio, es imposible resumir aquí.

Se recabó la edad, el nivel educativo y la clase social, aunque no se definieron como criterio de inclusión. Respecto del método de encuentro (virtual o presencial), entre las entrevistadas del Área Metropolitana de Buenos Aires (AMBA) se les dio a elegir qué medio preferían, mientras que a las participantes del interior del país se les ofreció una entrevista virtual, dado que las investigadoras residen en AMBA. La mayoría de las entrevistas de AMBA se realizaron de manera virtual por elección de las participantes, aunque dos fueron presenciales en el domicilio o lugar de trabajo de la entrevistada. Todas las entrevistas fueron grabadas y transcriptas para su análisis. 

El total de las 18 personas entrevistadas eran mayores de 40 años, cisheterosexuales y estaban casadas, de las cuales 16 participantes tenían 1 hijo o hija por gestación por sustitución, aunque una había adoptado un hijo o una hija anteriormente a la gestación por sustitución, y otra tenía dos hijos o hijas por gestación por sustitución. Las niñas o los niños que habían nacido por gestación por sustitución eran menores de cinco años al momento de la entrevista. La mayoría de las entrevistadas habitaban en AMBA, aunque un porcentaje menor eran del interior del país, especialmente la zona centro. 

Todas las mujeres entrevistadas para este estudio fueron informadas de los objetivos, el tratamiento dado a la información, y tuvieron posibilidad de evacuar sus dudas antes de comenzar la entrevista. Todas fueron comunicadas de su derecho a no contestar determinadas preguntas o a suspender la entrevista en cualquier momento, si lo desearan. Como es de rigor en este tipo de investigación, los nombres propios, nombres de ciudades, de clínicas, de profesionales médicos, de parientes, y cualquier otra información que pudiera develar la identidad de la persona entrevistada ha sido omitida y/o sustituida, con el objetivo de preservar la confidencialidad. Este estudio fue aprobado por la Comisión de Ética en la Experimentación Animal y Humana (CEEAH) de la Universitat Autònoma de Barcelona con número de referencia CEEAH 5974. 

## RESULTADOS

### Gestación por sustitución en Argentina y Ucrania

En Argentina, el acceso a las técnicas de reproducción humana asistida está regulado desde el año 2013^17^ y desde el 2015 constituyen una nueva fuente de filiación^54^. El anteproyecto de reforma del Código Civil y Comercial propuso la inclusión de la gestación por sustitución en el artículo 562, hecho que fue discutido en audiencias públicas en 2012. El artículo estipulaba la autorización judicial previa y el consentimiento de las partes antes de la transferencia embrionaria^55^, y establecía que la justicia debía homologar ese consentimiento solo en caso de que la gestante no hubiera recibido retribución, entre otros aspectos^56^^,^^57^. 

Tras más de dos años de debate, la Comisión Bicameral decidió eliminar el artículo 562 del anteproyecto, argumentando la complejidad ética y jurídica del tema. Esta eliminación fue posiblemente efecto de la influencia de sectores conservadores y de la Iglesia Católica, y fue acompañada de la sugerencia de promover un debate interdisciplinario futuro^57^. Tras la eliminación del art. 562 original, la gestación por sustitución quedó sin legislar, ya que ella no está contemplada en la Ley de acceso a la reproducción asistida (26.862), aunque tampoco prohibida, por lo que ocupa un limbo regulatorio entre la legalización y la prohibición. Desde la sanción del nuevo Código Civil y Comercial que finalmente no legisló esta práctica, se han presentado diferentes proyectos de ley, sin éxito^58^^,^^59^^,^^60^^,^^61^. 

El Código Civil y Comercial sancionado en 2014, en el nuevo artículo 562 sobre la voluntad procreacional -central para la disputa jurídica- establece que quienes nacen por técnicas de reproducción humana asistida son hijas e hijos de quien dio a luz y del hombre o de la mujer que *también* ha prestado su consentimiento previo, informado y libre, complicando aún más el escenario de una posible legislación de gestación por sustitución, en la medida que entiende que la maternidad se establece a través del parto. Sin embargo, en casos de gestación por sustitución, mientras que la persona gestante atraviesa el parto, no ejerce la maternidad ni tiene voluntad procreacional. Y mientras que varios fallos han argumentado sobre la inconstitucionalidad de este artículo en la jurisprudencia reciente^19^^,^^61^, el último fallo de la Corte Suprema de Justicia de la Nación, en el mes de octubre de 2024, determinó que la persona que da a luz debe ser considerada legalmente como madre. Además, el fallo rechazó la posibilidad de que el artículo 562 pueda ser considerado anticonstitucional, aún en los casos en que la mujer gestante no presente voluntad procreacional como ocurre en la gestación por sustitución, exhortando en su lugar al Congreso de la Nación para que sancione una legislación específica para estos casos. Estas novedades legislativas complican actualmente aún más el panorama para la realización de gestación por sustitución en Argentina.

La falta de regulación específica hace que, para quienes realizan gestación por sustitución en el país (ya sean extranjeros o nativos), sea necesario recurrir a la justicia de manera de legalizar el proceso. En la mayoría de las provincias del país (excluyendo a la Ciudad Autónoma de Buenos Aires), uno de los recursos jurídicos más utilizados es la autorización judicial -previa al inicio del procedimiento o posterior al embarazo y antes del nacimiento- solicitada por las personas interesadas en llevar adelante un arreglo de gestación por sustitución, siendo en general este pedido respondido de manera afirmativa^62^. Hasta la fecha, se han emitido más de 80 sentencias, muchas de ellas favorables (cinco de ellas pendientes de resolución en la Corte Suprema de Justicia) a través de las cuales el ámbito judicial aloja la figura de la gestación por sustitución.

En el caso de la Ciudad Autónoma de Buenos Aires, estuvo vigente desde 2017 a junio de 2024 la disposición 93/2017 -modificada por las disposiciones 103/2017 y 122/2020- del Registro Civil que permitía la inscripción preventiva de los niños y las niñas que nacían por gestación por sustitución *sin la judicialización de los casos*. Esta disposición fue el resultado de una puja política entre algunos sectores del activismo reproductivo y el activismo LGTB y, durante su vigencia, implicó que muchas personas que buscaban realizar gestación por sustitución en Argentina pudiesen llevar a cabo el procedimiento, allanando la falta de regulación. Debido a las garantías legales que otorgaba, esta disposición tuvo como un efecto tal vez no buscado que muchas personas se mudasen a este ámbito geográfico y, más inquietante aún, lograran hacer mudar (al menos temporariamente y durante el período cercano al parto) a la gestante, con el objeto de poder realizar la inscripción preventiva en el Registro Civil de la Ciudad Autónoma de Buenos Aires. Debido a esta razón y otros efectos, como el incremento en el turismo reproductivo derivado del hecho de que quien nacía de gestación por sustitución en la circunscripción podía inscribirse sin problemas a nombre de las personas progenitoras intencionales, incluso si la transferencia del embrión se había realizado en otro país^61^, sumado a algunas denuncias por trata de personas, la disposición fue finalmente dejada sin efecto en 2024. En conjunto, con el reciente fallo de la Corte Suprema de Justicia de la Nación ya mencionado, la anulación de esta disposición configura un panorama adverso a la realización de gestación por sustitución en Argentina.

En vistas de esta ausencia normativa y de la incertidumbre legal que se deriva de ella, muchas personas que lo necesitan deciden no realizar gestación por sustitución en el país. Un destino común para los sectores de clase media y media alta, que buscan alternativas a la gestación por sustitución en Argentina, es Ucrania, un país que ha regulado el acceso a la gestación por sustitución para parejas heterosexuales casadas y en las cuales alguno de los dos progenitores pueda mantener un vínculo genético con el bebé (es decir, que la madre o el padre de intención estén en condiciones de usar sus propios gametos)^3^^,^^5^​​. La gestación por sustitución comercial se desarrolló en Ucrania a partir del cambio de milenio, y este país forma parte -junto con México^28^^,^^39^^,^^51^, Tailandia^37^ y, hasta hace unos años, India^12^^,^^49^^,^^63^^,^^64^- de un conjunto de destinos donde la gestación por sustitución es legal, pero a costos más bajos que en otros países, como EEUU^3^^,^^33^^,^^50^^,^^65^.

Las parejas argentinas que realizan gestación por sustitución en Ucrania suelen recurrir a esta técnica luego de haber intentado procrear naturalmente y de haber realizado muchos tratamientos reproductivos, sin resultados. La mayoría de estas parejas relata haber atravesado situaciones reproductivas extremadamente difíciles antes de recurrir a la gestación por sustitución, entre las que se cuentan abortos espontáneos repetidos, el nacimiento de un hijo o hija que murió al poco tiempo o nació sin vida, la pérdida de partes reproductivas funcionales como el útero o los ovarios, la posesión de formaciones orgánicas no estándares de los órganos reproductivos que impiden la reproducción, o el haber enfermado gravemente antes, durante o como consecuencia de la realización de tratamientos reproductivos (con cáncer, enfermedades autoinmunes, enfermedades de la sangre, síndrome de hiperestimulación ovárica, etc.). También, la mayoría de las parejas que realizaron gestación por sustitución en Ucrania entrevistadas para esta investigación habían intentado adoptar niños o niñas en Argentina, también sin resultados. 

En Kiev existen varias clínicas privadas que realizan gestación por sustitución para personas extranjeras, algunas más conocidas que otras. Según los relatos obtenidos, las clínicas cuentan con un “Departamento de habla hispana” a cargo de una coordinadora ucraniana que habla español. Las comitentes mantuvieron sobre todo con esta coordinadora el contacto antes y durante la gestación por sustitución. Si bien no todas las participantes de este estudio realizaron la gestación por sustitución en la misma clínica, la mayoría de las entrevistadas puso de manifiesto estar muy satisfecha con el servicio que recibió de parte de la clínica. Una vez que en la clínica ucraniana recibían los estudios diagnósticos argentinos, se programaba ya sea la obtención de la muestra de semen del varón de la pareja, la extracción de los óvulos de la futura madre y/o la selección de la donante. En la mayoría de los casos relatados para esta investigación, la muestra de semen fue obtenida en Kiev, aunque en alguno de los casos se obtuvo un permiso legal especial para que la muestra fuese enviada, en lugar de que el varón tuviese que viajar a dejarla, debido al comienzo de la guerra en Ucrania. En la mayoría de los casos, las entrevistadas no utilizaron sus propios óvulos debido a problemas en su producción, a fallas previas reiteradas de implantación utilizando sus óvulos, a enfermedades preexistentes, etc. 

### Conocerla o no conocerla, esa es la cuestión

En esta sección indagamos en los deseos que expresaron haber tenido las entrevistadas de este estudio respecto de la posibilidad o no de conocer a la gestante *en persona* una vez que llegasen a Ucrania para recibir al bebé, así como en las implicancias que estas expectativas tuvieron para la comprensión y experiencia del período gestacional en sí. Lógicamente, estos momentos han sido conceptualmente reconstruidos sobre la base de la información recuperada en las entrevistas. Se trata, por lo tanto, de momentos “ideales” que nos permiten observar analíticamente el despliegue de la relación (o la renuencia a entrar en un vínculo) de las gestantes y las comitentes, desde el punto de vista de estas últimas.

En la mayoría de los casos, las comitentes indicaron que habían querido conocer a la gestante una vez llegadas a Ucrania para encontrarse con su bebé; algo que no siempre es posible ya que también se consulta a la gestante si desea conocer a las personas comitentes. Por ejemplo, en el caso de Paola, ella contó que:

I: ¿*Y vos la querías conocer a* [Nombre de la gestante]?

P: *Sí, yo la quería conocer y me hizo muy bien conocerla, porque los ucranianos son muy correctos, pero son muy fríos, por ejemplo, a mí la médica que me atendió todo el proceso de la estimulación ovárica, y la extracción de los óvulos, nunca hizo contacto visual conmigo, no me miraba a los ojos la médica, entonces como que yo quería conocer a esa persona que llevó nueve meses a mi bebé, y cuando la conocimos, una persona tan dulce, tan dulce, con una mirada tan dulce, ella siempre nos miró a nosotros como con este gesto, y a pesar de que no entendía nada y que las coordinadoras traducían, ella siempre como así, nos miraba con mucho amor, y a mí eso me dio mucha paz y mucha tranquilidad y me dio la certeza de que ella había cuidado a nuestra bebé y que la había amado esos nueve meses también.* (Paola)

Como parece indicar Paola, conocer a la gestante era importante para ella no solo porque llevó a su hija en su panza durante nueve meses, sino también porque sus experiencias previas con la cultura ucraniana le habían mostrado que “los ucranianos son correctos, pero muy fríos”, por ejemplo, como la médica que le extrajo los óvulos ya que, en su caso, para el procedimiento de gestación por sustitución, utilizó sus propios óvulos. Para ella, conocer a la gestante parece tener que ver entonces con poder entablar, al menos transitoriamente, un tipo de relación distinto a aquel que caracterizó encuentros previos con la cultura ucraniana; en particular, con poder establecer un vínculo menos distante, más afectuoso e, incluso, connotado por sentimientos como el amor. Paola destaca la “mirada tan dulce” de la gestante, que se trasluce incluso a pesar de las barreras idiomáticas; es decir, un vínculo que no necesita palabras. Este encuentro, entre Paola y su gestante, opera también como garantía de que la bebé había sido amada durante su gestación; es decir, que el vínculo amoroso entre ella y la gestante se ofrece como prueba de las condiciones (amorosas) en que ocurrió la gestación.

En pocos casos, sin embargo, la comitente indicó que no había deseado conocer a la gestante. Es, por ejemplo, el caso de Marianela, quien consultada sobre las razones por las cuales no quiso conocerla, dijo que:

*Yo asumo que en mi caso se trataba de no tener muy definido el rol de ella, como que lo veía como un no sé, como un obstáculo, por llamarlo de un modo, lo que fuera, entre mi hijo y yo* […] *Es un tema concreto de que yo no tenía definido el rol. Hay muchas parejas que tienen contacto, que se mandan fotos, de hecho, en esa época se podían ver las ecografías por zoom… ahora creería que ya no, pero bueno, en esa época mucha gente las veía y yo no quería, quería que me mandaran los resultados de la ecografía y nada más, porque yo creo que sentía bueno eso, como que no quería tener mucho contacto. Y por otra parte decía “bueno, ¿y si no me gusta y pienso todo el tiempo* [que está] *en la panza de alguien que no me cae bien?”. Y de hecho el día que la conocí* [fue] *en el contexto en que fue a firmar la renuncia a los derechos y a percibir la última cuota. De hecho, yo le pregunté* [a la coordinadora]*, le digo “no puedo no verla, no sé qué…”. Dijeron: “bueno podemos hacer…” y yo al final dije: “bueno, está bien, la veo”*. […] *Entonces vamos, y cuando entramos, uno* [de los padres por gestación por sustitución] *dice: “ah, sí, esta es la mía no se qué…”. Y estaba la gestante de las parejas, una cosa desagradabilísima, una mujer así, alta, grandota, con un tapado de astracán, unas uñas así de largas, y el padre bueno le da el dinero y le pregunta: “¿cómo estás?*”*. Pensá que era toda gente que había parido hacía una semana. La tipa lo miró así, contó la plata, se la guardó en la cartera y se fue. Y yo pensaba “claro, imagínate si me hubiera tocado a mí eso desagradable*”. *Entonces, después no, la gestante de Nicolás resultó ser una chica divina, gordita y nada… de hecho le había… le bordó, allá es muy típico hacer bordados con mostacillas y le bordó como unos adornos de arbolito de Navidad y dijo: “esto lo hice mientras estaba embarazada para Nicolás” y me lo trajo de regalito, no, no, divina*... (Marianela)

El relato de Marianela permite observar las razones que ella le adjudicaba a no haber querido conocer a la gestante ni durante ni luego del embarazo (aunque finalmente, por casualidad, la conoció). Para ella, la indefinición respecto de qué rol ocupaba y/o ocuparía la gestante en la vida de ella y de su hijo era parte de los motivos para no querer conocerla. Adicionalmente, Marianela sentía miedo de que, si al conocer a la gestante no le caía bien, se quedara preocupada porque su hijo había sido gestado por alguien con quien no tenía afinidad. Este relato muestra que, al igual que en el caso de Paola, lo que pasa durante el embarazo, en qué estado emocional ese embarazo fue llevado, y quién había sido, en definitiva, la gestante, son aspectos de suma importancia para las comitentes. De hecho, la posibilidad de una disociación o desconexión de algún tipo (emocional y/o cultural) entre la gestante y la comitente, a la que refiere Marianela a través del término “miedo”, parece haber sido real. Marianela relata, en efecto, haber conocido a otra gestante, no la de su hijo, a quien caracteriza de “cosa desagradabilísima” por estar aparentemente solo preocupada por el dinero, no interesarse por el vínculo con las personas comitentes y, para peor, utilizar un tapado de astracán (es decir, de piel de cordero no nacido o recién nacido); hecho cuyas oscuras connotaciones simbólicas no se le pierden a Marianela. Sin embargo, en su caso particular, y al haber conocido finalmente a su gestante casi de casualidad, Marianela pudo tener la certeza de que *su* gestante había sido “una chica divina”, que además le había bordado una decoración navideña recordatoria para su hijo Nicolás.

El caso de Ivana ilustra aspectos semejantes. Ivana no había querido tampoco conocer en persona a la gestante antes y durante el embarazo. Sin embargo, al igual que Marianela, finalmente la conoció, en un cambio de decisión rápido, al parecer influido por las circunstancias:

I: ¿*Con la gestante entraste en contacto*?

I: *No, yo no quería conocerla* [...] *Y cuando a mí me lo entregan, Ciro nació el 13 de enero y me lo dieron el 15 de enero. El 17 de enero teníamos que ir a hacer el estudio de ADN, y ese día nos pasan a buscar por el departamento donde estábamos parando y me dice la traductora que la gestante se iba hoy, ese día se iba de Kiev porque no era de Kiev, era de un pueblito, de un campo, y que si nosotros queríamos, ella estaba disponible para conocernos y conocerlo a Ciro, porque ellas no los conocen a los bebés* [...] *Eh, bueno, nos dicen así, me dice Sebastián: “es tu decisión, yo no tengo problema, pero decidilo vos a esto”. Y entonces le dije: “bueno, si ella no tiene problema, vamos”. Y entonces fuimos a hacer el ADN y de ahí, eh, fuimos a la clínica donde estaba ella* [...] *Y era una chica jovencita, no sé si tendría 30 años. Se ve que era muy humilde. Divina, me mostró sus hijos* [...]. *Y fue así, yo estaba sentada ahí con Ciro y entró ella, era una sala grande, flaquita, así chiquita y se vino así derecho a abrazarme. Y yo ahí lloré lo que no había llorado en 15 años, calculo, 16. Y fue, creo que mejor gestante para Ciro no había* [llora]. *Me tocó la mejor. Un ángel. Un ángel. No tengo dudas de que lo cuidó… me dijo que ella hacía el tratamiento más que por el dinero que recibía, porque ella quería que las personas que querían ser madres no se queden con las ganas* [llora]. [...] *Y que sabía que esta vez había ayudado a alguien muy especial y que le bastaba con verme.*

I: *Qué lindo lo que te dijo*.

I: *Sí. Y bueno, me mostró sus hijos, y se fue. Me dejó una manta tejida por ella* [...]

I: ¿*Y vos le regalaste algo*?

I: *Yo no le había llevado regalo porque como no pensaba conocerla…* [...] *yo le había dicho a Sebastián que yo no quería conocerla porque pensé que no lo iba a soportar. Tenía miedo.*

I: *¿Qué pensabas si la conocías?*

I: *Eh… Yo tenía miedo por Ciro, porque yo dije: “¿Y si Ciro le reconoce la voz y piensa que lo estamos sacando del lugar donde por ahí tendría que estar naturalmente?”. Y Sebastián me... Sebastián es muy, no es trágico como yo. “Eso pensás vos” me dice, “yo no creo que pase eso”*.

I: *Claro*.

I: *Y no, no, ni bola le dio Ciro, pobre. Me preguntó si lo podía alzar y se lo di antes de que termine de preguntármelo. Pero él, nada* [hace un gesto como que a Ciro ni le importó]. *Fue algo muy lindo, pero muy lindo.* (Ivana)

Al igual que en los dos casos anteriores, la historia de Ivana resalta, por un lado, cuán importante parece haber sido para las entrevistadas quién era la gestante y cómo se imaginaban que llevaría el embarazo. Esto es cierto tanto para quienes deseaban conocer a la gestante de sus hijos o hijas, como para quienes no querían conocerla, por lo que la asunción de que existe una conexión entre el tipo de persona que es la gestante y cómo llevó el embarazo es independiente de las expectativas de conocimiento. Este punto ilustra que, al menos desde la mirada de las comitentes, antes que un mero “receptáculo”, “incubadora”, “horno” u otro tipo de apelativos negativos para referir a la gestante, con los que se suele criticar a la gestación por sustitución^15^^,^^66^, existe una presunción en las comitentes de que *quién es la gestante importa para el tipo de embarazo que esta lleve* (“*Me tocó la mejor. Un ángel* [...] *No tengo dudas de que lo cuidó*”). Para ellas, que la gestación haya ocurrido con amor, que la gestante haya tenido “*una mirada tan dulce*” o que haya demostrado ser “*divina*”, “*un ángel*” y no solo interesada en el dinero, pone de manifiesto que estas comitentes están lejos de concebir a estas mujeres como *meras* gestantes de sus hijos o hijas.

Al mismo tiempo, también resulta importante detectar que estos relatos hablan de relaciones entre gestantes y comitentes marcadas por ciertos momentos de reciprocidad, consideración mutua, cordialidad e incluso afecto. En el relato de Ivana, cuando finalmente y a contrapelo de sus deseos originales la conoció, la gestante le mostró a sus hijos en fotos, “*se vino derecho a abrazar*[la]”, le dejó de recuerdo una manta tejida por ella, le dijo que había ayudado a alguien “*muy especial*” y le pidió alzar a Ciro, el bebé que había gestado. De la misma manera, la gestante de Marianela le bordó un árbol de navidad a su hijo, que le dejó como recuerdo. Como profundizaremos en la sección que sigue, estos intercambios distan mucho de hablar de relaciones puramente interesadas o instrumentales, dando cuenta por el contrario de elementos que permitirían caracterizarlas como, hasta cierto punto, mutuamente recíprocas.

### Fotos, videos y canciones: dispositivos de vinculación durante el embarazo

En esta sección analizamos de qué manera las entrevistadas de este estudio relataron haber mantenido relación con la gestante ucraniana durante el embarazo, mientras ellas estaban en Argentina. Prestamos especial atención al rol que ejercen lo que llamaremos “dispositivos de vinculación”, dentro de los cuales cabe incluir fotos, audios, videos, ecografías, cartas o bordados, entre otros. Nuestro interés está, en primer lugar, en dar cuenta de las maneras en las que estos dispositivos son utilizados no solo para entablar y mantener vínculos entre comitentes y gestantes, sino también entre gestantes y bebés, y entre bebés y comitentes. Por esta razón, en segundo lugar, buscamos considerar de qué maneras tales dispositivos no son *meros medios* de vinculación, sino auténticos *productores* de nuevos tipos de relaciones entre actores también nuevos (como es la gestante no madre, y el bebé gestado por gestación por sustitución)^2^. 

Esta indagación continúa una línea de trabajo que se ha focalizado en el rol de los dispositivos clínicos (como consentimientos informados, formularios de coordinación fenotípica y fotos) en la producción de filiación y desafiliación en la clínica de fertilidad^67^^,^^68^. A su vez, nos nutrimos de las categorías propuestas por Bruno Latour^69^ para caracterizar los dispositivos de vinculación como mediadores en tanto “transforma[n], traduce[n], distorsiona[n] y modifica[n] el significado de los elementos que se supone que portan”, a diferencia de los *intermediarios*, que “transporta[n] significado o fuerza sin transformación”^69^. A través de estos conceptos, entendemos que los dispositivos de vinculación tienen un efecto productivo y transformativo de los vínculos entre comitentes y gestantes, no siendo meramente un *medio* de conexión. 

Por lo general, las clínicas reproductivas que llevan a cabo los tratamientos en Ucrania suelen establecer contractualmente que el contacto entre gestantes y comitentes durante el embarazo ocurre solo a través de la clínica. Según algunas entrevistadas, este arreglo busca desalentar contactos informales, sobre todo para prevenir potenciales abusos (especialmente solicitudes de dinero extra al pautado o “chantajes”) de parte de las gestantes. Sin embargo, más allá de este contrato, la mayoría de las entrevistadas de este estudio tomó contacto de manera informal con la gestante durante el embarazo, ya sea por iniciativa propia o de la gestante o, de forma excepcional y debido al inicio de la guerra Rusia-Ucrania, a través de la propia clínica. En este estudio no observamos diferencias significativas en las relaciones entabladas cuando estas estuvieron mediadas por la clínica o cuando fueron directas entre comitentes y gestantes. En los casos en que las clínicas facilitaron los contactos de las gestantes a las comitentes esto se debió a que había comenzado la guerra en Ucrania, y a la dificultad adicional para mantener el vínculo con las gestantes debido a la interrupción de los sistemas de comunicación y a la disrupción del transporte, y con el objetivo de que las comitentes también ayudaran a monitorear el embarazo y asistir, dentro de lo posible dada la distancia, a las gestantes, muchas de ellas aisladas en sus localidades y, a veces, sin posibidad de acceder a medios de comunicación durante los bombardeos y/o evacuaciones. En este apartado no analizamos estos casos, ya que sus particularidades los hacen susceptibles de un examen diferente.

El contacto entre gestantes y madres de intención fue mantenido usualmente por medios tecnológicos digitales como Facebook, y utilizando softwares de traducción, como Viber. Los audios, videos y fotos figuran de manera prominente en los relatos de las entrevistadas argentinas que realizaron gestación por sustitución en Ucrania. En ellos, estos dispositivos permiten transmitir contenidos sonoros y/o visuales que intermedian productivamente en la relación entre comitentes y gestantes. Por ejemplo, en el caso de las fotos, nuevamente Paola relató:

I: *¿Vos tuviste contacto directo con la gestante?*

P: *No, además también por la barrera del idioma. Los ucranianos no todos hablan inglés, y por ahí la gente más joven habla inglés, pero por ahí es como un inglés más limitado, entonces toda la comunicación era a través de nuestras coordinadoras de fertilidad de habla hispana. Nosotros le mandábamos fotos, videos, canciones, un montón de cosas y ella se lo enviaba… ella* [la coordinadora] *también nos mandó fotos de ella* [la gestante]*, de la panza, nosotros recibíamos las fotos y ella recibía nuestros videos, con nuestras fotos, nuestros mensajes, audios, o yo le mandé canciones si ella quería escuchar, para que ella las escuche y para que Azul las sienta.* (Paola)

En la cita precedente, Paola relata cómo el contacto con la gestante durante el embarazo, que era intermediado por la clínica, estuvo fuertemente marcado por el intercambio de materiales audiovisuales como fotos y videos. Es interesante notar que más que en una sola dirección (de la gestante hacia las personas comitentes, con fotos de las ecografías o de la panza), como sería tal vez esperable, dado el interés en conocer los pormenores del embarazo, el intercambio es bidireccional: tanto Paola recibió fotos “de la panza” como la gestante recibió videos de las personas comitentes. Es algo similar a lo que expresó Carla quien, si bien (por renuencia de la gestante) no prosiguió el vínculo con su gestante más allá del parto, no solo recibió fotos, sino que también remitió fotos de ella y su marido (a través de la coordinadora de la clínica) para que la gestante los conociera:

C: *Cada mes, perfecto, nos mandaban el reporte con las ecografías, todos los análisis que le hacían, una fotito. Yo le pedí una foto de la panza de la gestante y nos mandó la foto con ella, todo, así que fue muy lindo, al verla a ella la tranquilidad, ¿no? nos dio como una tranquilidad verla, ver su cara, yo pensé que me iban a mandar la foto de la panza y nada más, ¿no? Claro, no, mandaron todo y fue re lindo* [...]

I: *¿Y con la gestante tuviste algún vínculo o no?*

C: *No, solamente a través de la coordinadora de habla hispana. Le mandaba mensajitos, cómo estaba, cómo se sentía*.

I: *Ah, le mandabas mensajes a través de la coordinadora*.

C: *Fotos nuestras. Para que nos conozca también, porque ella nos mandaba su foto, ¿no?* (Carla)

Estos envíos parecen tener entonces una triple función. En primer lugar, en una función más *informacional*, operan claramente para mantener a las personas progenitoras de intención informadas sobre el derrotero del embarazo y funcionan como testimonios visuales de que aquello que ha sido contratado está de hecho ocurriendo. En segundo lugar, en lo que podríamos llamar una función *afectiva*, permiten a ambos lados del tráfico conocerse en situaciones cotidianas e ir entablando una relación a lo largo del embarazo, en la que el intercambio de materiales fluye *en ambas direcciones*, inhibiendo la configuración de las personas comitentes como meros receptores y la de la gestante como dadora: todos los actores involucrados se convierten en receptores y dadores de materiales que transmiten afecto, interés y cordialidad. De hecho, los audios y videos son enviados por Paola con la expectativa de que sean escuchados no solo por el bebé, sino también por la gestante: “*con nuestras fotos, nuestros mensajes, audios, o yo le mandé canciones si ella quería escuchar, para que ella las escuche*”. Y en el caso de Carla, ella no solo recibe las fotos, sino que también las envía, desarticulando el supuesto ciclo meramente instrumental^70^^,^^71^ en el cual quien paga tiene derecho a recibir y quien cobra tiene la obligación de entregar. Por el contrario, en estas historias, quienes pagan y son los portadores de la intención de filiación *también remiten*, y quien debe entregar (tiempo, gestación y un bebé) también recibe. No hay nada menos instrumental que entregar una foto de una pareja de comitentes a una gestante. Se trata de un intercambio que lejos de estar motivado por el ánimo de beneficio, obedece a una lógica que aquí llamaremos afectiva y que excede con mucho el supuesto ánimo *extractivo* que tendría la gestación por sustitución por parte de las personas comitentes. De la misma manera, incluso cuando Carla solo pide a la clínica que le envíen una foto “*de la panza*”, algo que podría entenderse como una expectativa puramente interesada que particiona el cuerpo de la gestante y focaliza solo en la parte relevante para sus fines (la panza), recibe por el contrario una foto de cuerpo entero de la gestante donde la panza es un elemento más. 

Una vez más, así como en la sección anterior observábamos que, independientemente de que quisieran conocer a la gestante o no una vez que nació el bebé, a las comitentes no les era indiferente quién había sido la gestante del embarazo, especialmente en lo relativo a su personalidad, aquí también observamos que a las comitentes no les era indiferente la relación que mantuvieran a la distancia con la gestante. Antes que meros receptores (de información, ecografías, reportes sobre el estado del embarazo, etc.), las y los comitentes agenciaban activamente en un vínculo con la gestante que, aún intermediado por la clínica, buscaba hacer presente en la vida de la gestante a los comitentes “*para que ella los escuche*” o “*para que nos conozca también*”, operando en una dimensión afectiva antes que instrumental. 

Por último, en su dimensión más productiva, estos dispositivos de vinculación operan también, en algunos casos, como formadores de vínculos de parentesco. En el caso de Paola, esto puede observarse en la expectativa de que sea el bebé en gestación quien escuche la voz de sus progenitores durante el embarazo, de la misma manera en que la escucharía si el embarazo hubiese sido llevado por la madre. A este respecto, es muy significativo el término que elige utilizar Paola para describir esta expectativa: no solo busca que su hija escuche su voz, sino que la “*sienta*”, explotando muy productivamente la polisemia del verbo en castellano “sentir” como una acción que remite a la posibilidad de escuchar, pero también de ser afectado emocionalmente. 

### “Para mí es como si fuera familia”: Las relaciones entre comitentes y gestantes luego del nacimiento

En este apartado presentamos el análisis de cómo las comitentes entrevistadas relatan haber proseguido (o no, aunque en la gran mayoría de los casos sí lo hicieron) el vínculo con la gestante luego del nacimiento de sus hijos o hijas y del retorno a Argentina. En la medida en que todas las entrevistadas que habían viajado a Ucrania para realizar la gestación por sustitución tenían hijos o hijas menores a cinco años, estos análisis tienen la limitación de examinar relaciones de corto desarrollo temporal entre comitentes y gestantes, restringiendo el conocimiento sobre lo que ocurre cuando se hace más lejano el nacimiento y cuando, como han mostrado algunos estudios, puede distanciarse el vínculo entre gestantes y comitentes^72^^,^^73^. Sin embargo, tal como indican resultados de otras investigaciones, en muchos casos el vínculo prosigue, incluso más allá de los 10 años, como parte del deseo de las gestantes de mantener relación con el niño o la niña que procrearon por gestación por sustitución^1^^,^^26^^,^^74^.

Todas las entrevistadas de este estudio se sentían profundamente agradecidas hacia las gestantes por su descendencia, y muchas dijeron continuar aún en vínculo con ellas y/o desear que esto fuese así por mucho tiempo más, en los casos en que sus hijos o hijas habían nacido hace poco tiempo. Aunque en este estudio no se realizaron entrevistas a gestantes, los relatos de las comitentes parecen indicar que, para muchas gestantes, la continuación del vínculo con los progenitores de intención y con los bebés que ellas gestaron también parece ser importante. Por ejemplo, Catalina relata que su gestante le pidió una foto del bebé, tal vez a modo de recuerdo de esa gestación, ya que en su caso no había podido tener contacto con el bebé inmediatamente luego del parto:

C: *A ellas no les permiten tener contacto con el bebé, según la maternidad en la que nazca, entonces* [la gestante] *me pidió si le podía mandar una foto* [del bebé] *a través de alguien que estaba internada en la misma clínica. Y después, una vez que ya nos dio el bebé y todo eso, ya nos intercambiamos teléfonos y tenemos una conversación* […] *te diría que cinco, seis veces en el año nos mandamos fotos, ella me manda de sus hijos, yo le mando de Mateo* [hijo] […] *no hay cumpleaños que ella no me mande un feliz cumpleaños para el nene, una relación de una persona que está a distancia, pero yo la aprecio muchísimo, no?*

I: *¿Qué sentís por ella?*

C: *No, mucho cariño, una persona que me ayudó, yo le digo “esto para mi es como si fuera familia”, te crea un vínculo*. (Catalina)

Como puede observarse en este extracto de entrevista, el rol de los dispositivos materiales de conexión como las fotos y los videos sigue siendo relevante para la (re)creación del vínculo una vez que la familia argentina retorna al país con su bebé. Como en el apartado anterior, vemos también que el tráfico de estos dispositivos de conexión es bidireccional (de las gestantes a las comitentes y viceversa) y nuevamente podemos observar en ello un uso que excede lo meramente instrumental: no se trata de conservar un vínculo o una foto de la gestante solo porque puede ser importante para el niño o la niña una vez que crezca, sino también *por el cariño y agradecimiento mismo que se siente por esa gestante*; asimismo *la gestante también solicita y recibe* fotos del niño o de la niña y su familia a medida que va creciendo, lo que hace suponer que el entramado afectivo entre ambas familias es mucho más tupido de lo que cabría suponer si se asumiera un vínculo puramente instrumental entre ambos. Como dice la misma Catalina, “*es como si fuera una familia*”; la familia misma se ha ampliado para contener a la gestante, las personas comitentes y su hijo o hija, e incluso la propia familia de la gestante: “*ella me manda* [fotos] *de sus hijos*”. Algo muy semejante aparece en la siguiente cita de la entrevista a Graciela, en la cual ella cuenta que mantiene contacto con la gestante aún hoy, algunos años después del nacimiento de su hijo:

G: *Súper divina y es el día de hoy que mantenemos contacto, y de hecho nos pasamos fotos, de ella, de su familia bueno, yo conozco a toda su familia, hablamos para los cumpleaños, para el último cumpleaños hicimos videollamada y se puso re contenta porque no se lo esperaba, en el bautismo de Oliverio hicimos una llamada con el sistema que estamos usando ahora nosotras, que lo hizo la madrina del nene porque yo no lo sé hacer, y nos contactamos con* [Nombre de la gestante] *y con mi hermana de España. Porque mi hermana* [que vive en] *España vino con nosotros a Kiev*. (Graciela)

Como puede verse en este extracto de entrevista, Graciela sigue encontrándose con la gestante de su hijo de manera remota a través de tecnologías digitales y, al igual que Catalina, lo hace sobre todo en los días importantes, como cumpleaños y bautismos. Nuevamente los dispositivos de conexión figuran prominentemente en este vínculo como aquello que permite sostener, pero también transformar, la conexión en lo cotidiano, y nuevamente, como en el caso anterior, ese tráfico es bidireccional (“*nos pasamos fotos*”) y habla de un vínculo que excede a la comitente, la gestante y al niño o la niña que se gestó. De hecho, Graciela conoce a toda la familia de la gestante a través de fotos, y la gestante conoce a la familia ampliada de Graciela, por ejemplo, a su hermana, con quien también se conectaron en el caso del bautismo de Oliverio. 

Es decir, las personas que se relacionaron a propósito de una gestación por sustitución siguen en contacto de diversas maneras, más allá del hecho puntual de la gestación: dedican tiempo y afecto a esas relaciones, y muestran un interés que no puede traducirse de ninguna manera a un intercambio monetario (que, por otro lado, ya ocurrió y tiene pocas probabilidades de repetirse). En la medida en que sería posible pensar que tanto la gestante como la comitente están unidas por fuertes sensaciones de agradecimiento (por haber gestado a su descendencia y por haber sido monetariamente retribuida y, en algunos casos, haber recibido de manera extra algunos regalos, respectivamente), está claro que los vínculos posteriores a los nacimientos, tal cual los hemos observado aquí, exceden con mucho la lógica del puro beneficio económico. Y mientras que no es posible descartar que el interés en un rédito monetario por parte de la gestante sea la causa principal o en algunos casos excluyente para la decisión de gestar para otros, se hace evidente en estos testimonios que, al menos en algunos casos, tales vínculos van mucho más allá de la lógica del *homo economicus*. En efecto, el mantenimiento y regeneración del vínculo a lo largo del tiempo y con posterioridad al nacimiento no busca ningún beneficio, no tiene un objetivo comercial. Caracterizar a estas relaciones como “instrumentales”, “interesadas” y puramente “comerciales” no es solo ofensivo: es etnográficamente impreciso y no se corresponde con la realidad viva de estas relaciones. Resulta difícil no ver en estos vínculos el carácter emergente de una relación singular que, si aún no tiene un nombre exacto en nuestra cultura, sí tiene claramente un conjunto de experiencias.

Nuestra posición es que esta hipótesis respecto del carácter no puramente instrumental o económico de estos vínculos se sostiene aún en los casos en que la relación entre comitentes y gestantes no continuó luego del nacimiento. Aunque nuestro estudio encuentra una limitación en que solo está basado en entrevistas con madres de intención y no con la gestante, la historia de Carla muestra que, a pesar de que el vínculo no prosiguió (tal como a ella le hubiera gustado), incluso para la gestante la gestación involucró “*algo más*” que puro dinero:

C: *Aparte, esto que, obviamente uno sabe que ella lo hace… ¿Cómo te puedo explicar? sabés que también es por necesidad y además tenés que tener un buen corazón y tener ganas de… y voluntad* [enfático] *de llevar a cabo… Aparte, ella me decía “decile que se quede tranquila”, eso me ponía* [la coordinadora], *“porque yo la cuido como si fuera un bebé mío. O sea, está super cuidada”. Y cuando salimos de la clínica yo le pregunt*é [a la gestante] *si quería tenerla a upa, despedirse, me dijo que sí, fue muy conmovedor, nos abrazamos y bueno, y yo le dije que la entendía, que siempre iba a estar en nuestros corazones y que le agradecíamos, pero… un agradecimiento eterno, por lo que había hecho, por ese acto de valentía* [enfático]. *Hay que ser valiente para hacer esto, hay que ser valiente y generoso*. (Carla)

En la interpretación de Carla, la gestante lo hace “*por necesidad*”, pero también por valentía y generosidad (“*hay que tener un buen corazón*”). Tal como muchos otros estudios han mostrado al analizar la simultaneidad de las motivaciones altruistas y económicas en la gestación por sustitución monetariamente recompensada^33^^,^^35^^,^^75^, esta madre explica las motivaciones de su gestante de esta doble manera. Pero más allá de sus propias interpretaciones, su relato deja entrever que para su gestante *también* hubo algo más allá del interés financiero. Por eso, la gestante de Carla aceptó despedirse de la bebé, aceptó ser abrazada por su comitente, protagonizando un último momento “conmovedor” al despedirse. Incluso, si este relato parece mostrar la prioridad del interés económico por sobre el solidario, impresión afianzada en la renuencia de la gestante a continuar con el vínculo luego del nacimiento, aún podemos detectar en él un *resto*, un “*algo más*”, un abrazo que se prolonga, la participación en un encuentro conmovedor con las personas progenitoras de la bebe que se gestó y que permanecerán siendo extraños, que habilita a cuestionar, una vez más, la dicotomía altruismo/economía como algo excluyente. 

## DISCUSIÓN

En este artículo hemos examinado las intenciones y experiencias de las comitentes argentinas de gestación por sustitución en Ucrania en lo referido a la posibilidad de mantener una relación con las gestantes de su descendencia. Dos grandes áreas de discusión emergen de nuestros resultados. Por un lado, la necesidad de ampliar y complementar el enfoque del feminismo liberal para la comprensión de la gestación por sustitución, en vistas de su carácter probadamente *relacional*. Por otro lado, nuestros datos y análisis permiten observar la improductividad de un acercamiento a esta problemática desde una perspectiva que sostenga la exclusión conceptual entre altruismo y comercio (y todas sus modulaciones semánticas afines: solidaridad, ayuda, favor; pago, interés, compraventa e, incluso, explotación, etc.).

En cuanto al primer punto, como han mostrado estudios previos^2^^,^^3^, si bien la gestación por sustitución es un tipo de arreglo reproductivo en el cual se pone en escena la disputa sobre la autonomía gestacional de la gestante y la autonomía reproductiva de las personas comitentes, que tienen como agente central de esos derechos al sujeto liberal, la gestación por sustitución es también un procedimiento en el cual las personas participantes se encuentran fuertemente entramadas. Los “enredos reproductivos”^25^ de la gestación por sustitución hacen pensar que el marco excluyente del feminismo liberal, que prioriza analíticamente el ejercicio de la autonomía individual para la valoración de las posibilidades de la gestación por sustitución^76^, es limitado para dar cuenta de las prácticas reales y concretas de la gestación por sustitución. Si bien la capacidad de libre elección gestacional de la gestante es central para su desempeño en esa función, es claro que cualquier proceso de gestación por sustitución supone la distribución de la agencia gestacional y reproductiva y el entramado de los actores en una serie de (nuevas) relaciones que hacen necesaria la asistencia complementaria de otro marco teórico más allá del feminismo liberal. En este sentido, nuestra postura empíricamente informada por nuestra investigación es que el feminismo liberal y el feminismo de la ética del cuidado^76^ no son contradictorios, sino que deberían ser complementarios en el enfoque político y teórico de la gestación por sustitución; porque mientras el primero enfatiza el ejercicio de la autonomía gestacional de la gestante y la autonomía reproductiva de la comitente, el segundo permite tematizar las relaciones existentes entre gestantes y comitentes y cómo estas deberían estar enfocadas bajo la lógica del cuidado mutuo. Quizá esta complementariedad no sería tan difícil de lograr en vista de lo arrojado por estudios como el que aquí se presenta, en el que es posible observar las relaciones de conocimiento, reciprocidad, interés e incluso afecto mutuo entre gestantes y comitentes que hemos rastreado a lo largo de nuestro análisis. 

En relación con el segundo punto que queremos establecer en esta discusión, la cuestión de la ineficacia de la dicotomía altruismo/interés, si bien es cierto que la gestación por sustitución, cuando no está regulada y se realiza en contextos de precariedad económica, como pueden ser ciertas zonas de Argentina o de otros países periféricos, puede dar lugar a prácticas abusivas y de explotación, tal como se está indagando actualmente en Argentina respecto de la posibilidad de la existencia de una red de trata de mujeres gestantes captadas localmente para ser contratadas por ciudadanos extranjeros^77^. En este sentido, consideramos importante enfatizar que no toda la gestación por sustitución es necesariamente una práctica explotadora. Como ya hemos destacado, los estudios socioantropológicos sobre gestación por sustitución tienen un largo recorrido en el campo de las investigaciones sobre reproducción, especialmente en el Norte Global. Estos antecedentes han aportado matices políticos, sofisticación teórica y análisis empíricos de las muy heterogéneas maneras a través de las cuales se realiza gestación por sustitución en diferentes lugares del mundo, complejizando la mentada dicotomía altruismo/comercio y mostrando que cuando es correctamente legislada, y aún en situaciones de intercambio monetario, la gestación por sustitución no excluye sentimientos de reciprocidad, solidaridad y ayuda que, en principio, la alejarían de la lógica de la explotación. Sin embargo, a pesar de este trabajo, la gestación por sustitución sigue siendo hoy negativamente caracterizada en ciertos activismos, cierto tipo de intervenciones teóricas, y en los medios de comunicación masiva. Ilustraciones de estas posiciones en el terreno de la psicología, la bioética y la teoría feminista son, por ejemplo, los trabajos de la psicóloga perinatal Elena Crespi^16^ y de la abogada, especialista en bioética y feminista TERF (*Trans-exclusionary radical feminist*) Nuria González López^15^, en los que la gestación por sustitución es caracterizada como un “negocio”. Mientras que los feminismos occidentales continúan divididos sobre la cuestión de la gestación por sustitución en dos posiciones principales (el feminismo liberal y el feminismo de la “ética del cuidado”), en Argentina, a pesar de la diversidad de posturas, prima una posición abolicionista o crítica de la gestación por sustitución entre los feminismos, principalmente, sobre los vectores de la instrumentalización, explotación y vulneración de la mujer gestante^78^^,^^79^. Esta orientación es congruente con la publicación en 2020 del “Manifiesto Latinoamericano contra la Explotación Reproductiva”^80^, al cual adhirieron una gran cantidad de organizaciones feministas de la región. En los medios de comunicación masivos, la gestación por sustitución sigue siendo representada en ocasiones a través de abordajes sensacionalistas (y a veces también filosóficos) que la caracterizan como una forma contemporánea de explotación y esclavitud^81^^,^^82^.

Mientras que consideramos muy valiosos los aportes y propuestas de los feminismos que se han preguntado por la posible vulneración de los derechos, autonomía y subjetividad de las gestantes^83^^,^^84^, nuestro trabajo muestra empíricamente que, al menos en el caso de las relaciones entre comitentes argentinas y gestantes ucranianas, el panorama es más complejo y no permite la representación de la gestación por sustitución solo como una forma de explotación, instrumentalización, cosificación o incluso esclavitud. Sin considerar que todas estas formas de observar no son idénticas, entendemos que todas ellas comparten el rasgo común de oponerse a la realización de la gestación por sustitución por considerarla una relación abusiva hacia la gestante, implicando la pérdida de su autonomía gestacional, y poniéndola en el lugar de un instrumento comercialmente intercambiado para la satisfacción de un deseo reproductivo de otra mujer o pareja. Sin embargo, y aunque nuestro estudio tiene la limitación de considerar, por ahora, solo la voz de las comitentes argentinas y no de sus contrapartes ucranianas, nuestros análisis permiten observar que no podríamos caracterizar la relación entre estas dos figuras como de pura “explotación” (o nominaciones afines). Tal como hemos mostrado, en los relatos que hemos relevado se refiere de manera muy frecuente al vínculo bidireccional de preocupación, cuidado y apoyo mutuo entre comitentes y gestantes. Nuestro análisis del uso de dispositivos de vinculación, como son los videos, las fotos, las canciones, los audios, etc., permite observar que estos no fluyen en una única dirección (desde la gestante a los comitentes, por la “*obligación*” de mantener informada a la pareja sobre el derrotero del embarazo), sino que, por el contrario, las personas se entraman en vínculos bidireccionales. Las comitentes relatan así haber enviado sus fotos, sus videos, sus audios, etc., a la gestante, “*para que ella las escuche*” y “*para que ella* [...] [los] *conozca también*”, y haber recibido fotos de la familia de la gestante, en un circuito que parece rehuir la simple caracterización de instrumentalidad. Asimismo, nuestros datos muestran también que los vínculos entre gestantes y comitentes, o la posibilidad de conocerse en persona, se iniciaron o produjeron, en muchos casos, por iniciativa de la gestante antes que de la comitente, lo cual desbarata la adjudicación de una posición subordinada y carente de agencia a la gestante. Como hemos mostrado, además, las relaciones entre gestantes y comitentes tuvieron una prolongación más allá del nacimiento del bebé, y esos vínculos se traducen en la conexión frecuente que tienen, por ejemplo, para eventos significativos como cumpleaños y bautismos de ambas familias. ¿Podría pensarse que las gestantes aceptan hacer videollamadas, enviar mensajes de saludos, o recibirlos, si hubieran sido mujeres explotadas, cosificadas e involuntariamente transaccionadas en una relación abusiva? ¿Podría de verdad sugerirse que esas gestantes mantienen esos vínculos posteriores a los nacimientos por razones de explotación, cuando ya han recibido su compensación económica y la gestación por sustitución, estrictamente hablando, ha llegado a su fin? No lo creemos. De la misma manera, si las gestantes hubieran entrado en vínculo con las comitentes solo por haber sido cosificadas a través de una transacción económica, ¿por qué entonces se dieron largos abrazos con sus comitentes, por qué pidieron conocer a los bebés que gestaron, por qué les dijeron a las madres de los bebés gestados que eran “*alguien muy especial*”, por qué protagonizaron momentos de encuentro y despedida de alta emotividad, por qué les tejieron mantas y bordados a las niñas y los niños que no maternarían, y se los entregaron a sus madres, para que los guardaran como recuerdo? No estamos proponiendo aquí que las relaciones entre gestantes y comitentes sean perfectamente simétricas, que las gestantes ucranianas no participen en los circuitos globales de gestación por sustitución en posiciones subordinadas estructuralmente dadas por sus condiciones subalternas en las escalas de acceso a bienes y servicios, y a través de posicionamientos subjetivos marcados por una serie de determinantes socioeconómicos como escaso acceso a salud, educación, ingresos, etc. Pero eso no puede de ninguna manera llevarnos a pensar que cuando entran en vínculos de gestación por sustitución lo hagan desde posiciones que son de pura explotación. Este análisis es una simplificación que deja sin efecto conceptual la evidencia etnográfica de la complejidad y mutualidad de los vínculos que establecen con sus comitentes, y una renuencia a comprender las maneras en las que ejercen situadamente, y muchas veces desde la vulnerabilidad, su autonomía gestacional.
